# Machine learning for prediction of diabetes risk in middle-aged Swedish people

**DOI:** 10.1016/j.heliyon.2021.e07419

**Published:** 2021-06-25

**Authors:** Lara Lama, Oskar Wilhelmsson, Erik Norlander, Lars Gustafsson, Anton Lager, Per Tynelius, Lars Wärvik, Claes-Göran Östenson

**Affiliations:** a CGI Inc., Stockholm, Sweden; b Region Stockholm, Center for Epidemiological Research, Stockholm, Sweden; c Karolinska Institutet, Dept of Molecular Medicine & Surgery, Endocrine and Diabetes Unit, Stockholm, Sweden

**Keywords:** Machine learning, Interpretable machine learning, SHAP, Risk screening, Type 2 diabetes, Individual healthcare plan

## Abstract

**Aims:**

To study if machine learning methodology can be used to detect persons with increased type 2 diabetes or prediabetes risk among people without known abnormal glucose regulation.

**Methods:**

Machine learning and interpretable machine learning models were applied on research data from Stockholm Diabetes Preventive Program, including more than 8000 people initially with normal glucose tolerance or prediabetes to determine high and low risk features for further impairment in glucose tolerance at follow-up 10 and 20 years later.

**Results:**

The features with the highest importance on the outcome were body mass index, waist-hip ratio, age, systolic and diastolic blood pressure, and diabetes heredity. High values of these features as well as diabetes heredity conferred increased risk of type 2 diabetes. . The machine learning model was used to generate individual, comprehensible risk profiles, where the diabetes risk was obtained for each person in the data set. Features with the largest increasing or decreasing effects on the risk were determined.

**Conclusions:**

The primary application of this machine learning model is to predict individual type 2 diabetes risk in people without diagnosed diabetes, and to which features the risk relates. However, since most features affecting diabetes risk also play a role for metabolic control in diabetes, e.g. body mass index, diet composition, tobacco use, and stress, the tool can possibly also be used in diabetes care to develop more individualized, easily accessible health care plans to be utilized when encountering the patients.

## 1 Introduction

### 1.1 Personalized healthcare

The health care sector needs better opportunities for individualized support for both the patient and the healthcare staff. Program 4D developed during 2012–2017 as a project focusing on type 2 diabetes (T2D) as a collaboration between Karolinska Institutet and Stockholm County Council that is in charge of most health care within the county. It included a process of screening for T2D, a standardized care process to support the healthcare staff, and a specific digital support for patients and healthcare professionals were developed. The functions specified in this project are now standard routine in e-health solutions and are implemented in commercially available solutions. In this and similar e-health solutions, healthcare professionals and the patient jointly set up a personalized interactive healthcare plan with individually tailored activities, where the patient's measurements and activities are reported.

The overarching aim of this project was to analyze factors influencing individuals to develop T2D and, if possible, to create supporting tools for health care personnel to aid the development of individual health care plans.

### 1.2 Type 2 diabetes

Diabetes mellitus is a chronic disease characterized by elevated blood glucose levels. Elevated blood glucose can contribute to metabolic problems and subsequent tissue damage in the body. T2D is in most people caused by a decreased insulin sensitivity on a background of an impaired insulin secretion, which in turn contributes to increased blood glucose levels [1].

In Sweden, about 400 000 people live with diagnosed diabetes [2], and with consequential complications resulting in a negatively affected quality of life. Most patients have T2D and it is estimated that at least an additional 200 000 adult persons have not yet diagnosed T2D and an equal number of adults have prediabetes [2]. Prediabetes is a condition, including both impaired glucose tolerance and impaired fasting glucose, with a high risk of developing T2D.

Diabetes care in Sweden needs a stronger collaboration between patients and different parts of the care and more resource-efficient pragmatic clinical research on new treatment alternatives. By combining the development of innovative, agile, and digital tools with internal and cross-professional networks we want to stimulate conversations, and facilitate recruitment of investigators and patients to clinical trials of diabetes. In addition, this may enable resource-efficient, pragmatic studies and follow up on care, prevention and research on diabetes. Thus, diabetes care can be made more individual, value and evidence based. Diabetes is a pilot area for developing both digital solutions and working methods for networks and collaboration between them. The digital tools and working methods can then be used in any other chronic disease therapy area.

## 2 Data source

### 2.1 The clinical sub-sample of the Stockholm Diabetes Preventive Program, SDPP

The people included in the baseline study were selected by a two step procedure [3, 4]. First, all men and women in the age range 35–54 years, living in five municipalities of the greater Stockholm area, Region Stockholm, were selected with mail addresses and telephone numbers by the national population register. About 11,000 persons, received a letter asking if they were willing to participate in the study with the primary aim to find factors that can increase or decrease the risk of developing type 2 diabetes. According to the approval by the Karolinska Hospital Ethics Committee, all selected men and women received a letter asking if they were willing to participate in the study with the primary aim to find factors that can increase or decrease the risk of developing type 2 diabetes. Those who wanted to take part in the study responded by signing a letter, and thereby also giving their written consent to participate. About ten thousand persons then received a first simple questionnaire focusing on family history of diabetes (FHD). The first question was if the respondent self has got diabetes. If so, this person was not included in further studies. Questions were also which biological relatives had a known diabetes, preferably diagnosed as T2D (or diagnosed at an age above 40 years). FHD was defined as having at least two 1^st^ degree relatives (parents or siblings) with diabetes, or at least three more distal relatives with diabetes. As many as 24% of all persons had FHD. These and persons without known FHD, about 8000 persons, were then asked to attend the clinical baseline study at a study center in their own municipality. Eventually, a group of about 4000 men and 4000 women with FHD and an equally sized group without FHD were involved in the further investigations. All those studies consisted of a detailed questionnaire about lifestyle, socioeconomic and psychosocial matters, along with measurements of plasma glucose and insulin in an oral glucose tolerance test (OGTT), glycosylated haemoglobin (HbA1c), blood pressure, weight, height and hip circumference. In the baseline study, T2D was diagnosed in 51 women and 66 men, and prediabetes in 219 women and 259 men (Table 1).

**Table 1:** Participants of the epidemiological study.

	Women	Men	Total
**Baseline (1992–1998)**			
All investigated	4821	3128	7949
NGT	4551	2803	7354
IFG	124	143	267
IGT	70	76	146
IFG + IGT	25	40	65
Prediabetes (all)	219	259	478
T2D (new)	51	66	117
**Follow-up #1 (2002–2006)**			
All investigated	3318	2360	5678
NGT	2817	1660	4477
IFG	237	331	568
IGT	94	100	194
IFG + IGT	68	98	166
Prediabetes (all)	399	529	928
T2D (new)	54	87	141
T2D (incident)	48	84	132
T2D (all)	102	171	273
**Follow-up #2 (2014–2017)**			
All investigated	2019	1323	3342
NGT	1308	712	2020
IFG	394	336	730
IGT	83	76	159
IFG + IGT	138	110	248
Prediabetes (all)	615	522	1137
T2D (new)	96	89	185
T2D (earlier)	153	237	285
T2D (all)	230	326	556

The table summarizes the number (n) of individuals participating in the three investigations with a mean of 8–12 years apart and how they were diagnosed according to glucose tolerance.

NGT, normal glucose tolerance, IFG, impaired fasting glucose, IGT, impaired glucose tolerance, Prediabetes, sum of those with IFG, IGT and IFG + IGT, T2D new, diagnosed at the investigation and T2D incident, reported and ascertained diagnosed during interval between two investigations, e.g. the baseline and the Follow-up #1.

A 1^st^ follow-up study was carried out 8–10 years later, and a 2^nd^ follow-up about 20 years later, with at least 70% participation (Table 1). At the 1^st^ follow-up, we found 102 women and 171 men with T2D, and 399 women and 522 men with prediabetes, and at the 2^nd^ follow-up 230 women and 326 men with T2D and 615 women and 522 men with prediabetes. Those with diagnosed T2D at the baseline and the 1^st^ follow-up were not called to follow-up later, but received a letter asking them to fill in the questionnaire and also inform if any new relatives had been diagnosed with diabetes. Thereby, questionnaire responses were validated by comparing how the same questions were answered at three times. With time, i.e. from basline from the 2^nd^ follow-up, the percentage of persons with FHD increased from 50 to 57%, most likely due to ageing of relatives and increasing opportunities for everyone of diagnosis of diabetes in the primary care.

### 2.2 Data transformation

SDPP data from the baseline and the 10-year follow-up was used to predict a diabetes diagnosis in the investigations following 10 years later [3, 4]. The target variable to be predicted in the ML algorithm - consisting of WHO 1999 classification of diabetes [5] - was set to 1 if the person developed prediabetes or T2D during the follow-up and set to 0 otherwise.

The SDPP data containing the result from measurements and questionnaire answers were restricted to socioeconomic and psychosocial stress factors, physical and blood measurements along with self-estimated physical activity, dietary information and tobacco use. Factors or features, i.e. input variables to the ML algorithm, with a high amount of missing values were excluded from the dataset. The dietary features were set to categories with values in an increasing order from 1 to 8 depending on consumption frequency. Features with few missing values were not excluded, instead the missing values were replaced with the features median. The features included are listed in Table 2.

**Table 2:** Factors included in analysis.

**Factors increasing diabetes risk**
Heredity i.e. family history of diabetes
High age
High waist-hip ratio
High BMI
Systolic blood pressure increased
Diastolic blood pressure increased
Low physical activity
Male gender
**Factors decreasing diabetes risk**
Exercise
Higher socioeconomic strata
Lower age
**Factors not influencing diabetes risk in this study**

Tobacco use, cigarettes
Snus, oral moist tobacco
Chest pain, angina
General health
Psychologic distress:
Depression
Nervousness
Fatigue
Lethargy
Insomnia
Coffee

Since the machine learning algorithm only takes numerical values, features as gender was set to 0 for men and 1 for women. The feature for heredity (FHD) was set to 0 and 1 for persons without and with T2D in the family, respectively.

The dataset was split in training, test, and validation sets. The validation set was used to optimize the model hyperparameters using grid search and cross validation. The training and the test sets were used to train and evaluate the final model.

## 3 Method

In this section, the method and model evaluation are described. A Random Forest classifier [6] was trained to predict whether an individual develops prediabetes or T2D after 10 years of the measurement using binary classification. An interpretable machine learning model, SHAP TreeExplainer, was used to interpret the predictions of the Random Forest classifier. An ensemble of ML models was used to increase the robustness of the predictions and interpretations. A new measure is introduced for assessing the hyperparameter optimization of the model according to the robustness and accuracy of the model.

### 3.1 Model selection

The choice of ML classifier was made regarding the choice of possible interpretable ML models. There are interpretation models that can interpret any model, such as SHAP-KernelExplainer [7, 8] and LIME [9]. These types of models generally make the requirement to provide reference data. This is also the case with the method Integrated Gradients - which can only be used on neural networks [10]. Since it is desirable to minimize our impact on the model outcome, we want to avoid providing the interpretation model with reference data. We therefore avoid using this type of interpretation models.

Methods that do not require reference data are SHAP-TreeExplainer, expected gradients [7, 11, 12] and linear regression. Linear regression was excluded for several reasons; the model is sensitive to correlation between features and not complex enough to represent non-linear data. Expected gradients are applied to neural networks and TreeExplainer to tree models. Since the amount of data in SDPP is limited to about 8000 people, and with limited computing power, neural networks were excluded. That leaves us with SHAP-TreeExplainer, which can be applied to tree-based models.

In SHAP-TreeExplainer, SHAP values are calculated on leaf-level in the tree models. Estimating SHAP values, as proposed by Lundberg and Lee, is a unified measure of feature importance across various methods (11). It is however difficult to compare SHAP values between different tree models on leaf-level that do not use the same calculation methods. For instance, the SHAP values differ between models that use logarithmic odds instead of values between 0 and 1. This is the case for the gradient boosted decision trees; XGBoost [13], LightGBM [14], and CatBoost [15], that calculate SHAP using logarithmic values. These are difficult to transform into a 0–1 scale, due to that the relationship between the attributes is difficult to maintain without changing the source code in the SHAP library. This limits us to tree models that do not use logarithmic odds, such as Random Forest [6].

### 3.2 Model optimization

To optimize the hyperparameters of the ML model, grid search and cross validation were used [16]. The cross validation was done with 5 splits of the data in the grid search. The optimal hyperparameters were evaluated using “Area Under the Receiver Operating Characteristic Curve”, AUC [17], combined with a measure of robustness of the SHAP-values. To equally weight their influence, a measure, *S*, was proposed in the equation below.

To calculate the robustness, a measure $X_{ijkl}^{\prime }$ is firstly defined in the equation below, where $X_{ijkl}^{\prime }$ is a tensor of SHAP values per person *i*, feature *j*, cross validation split *k*, and parameter set *l*.

The tensor $X_{ijkl}$ is standardized to zero-mean unit-variance according to following equation

$${\text{X}}_{\text{ijkl}}^{\text{′}}=\frac{{\text{X}}_{\text{ijkl}}-\text{μ}\left({\text{X}}_{\text{ijkl}}\right)}{\text{σ}\left({\text{X}}_{\text{ijkl}}\right)}$$

where  μ is the mean and σ is the standard deviation.

The standardized tensor, $X_{ijkl}^{\prime }$, is used to calculate the robustness, Θ, for each tested parameter set *l* defined according to the following equation

$${\text{Θ}}_{\text{l}}=\text{μ}\left(\text{σ}\left({\text{X}}_{\text{ijkl}}\right)\right)$$

where Θ is a vector with one value per parameter set l.

The combined measure, *S*, of robustness and AUC per parameter set is defined according to the equation

$$S_{l}=AUC_{l}^{val}\cdot \left(1-{\text{Θ}}_{l}\right)$$

## 4 Results

### 4.1 Optimization of model parameters

**Figure 1:**
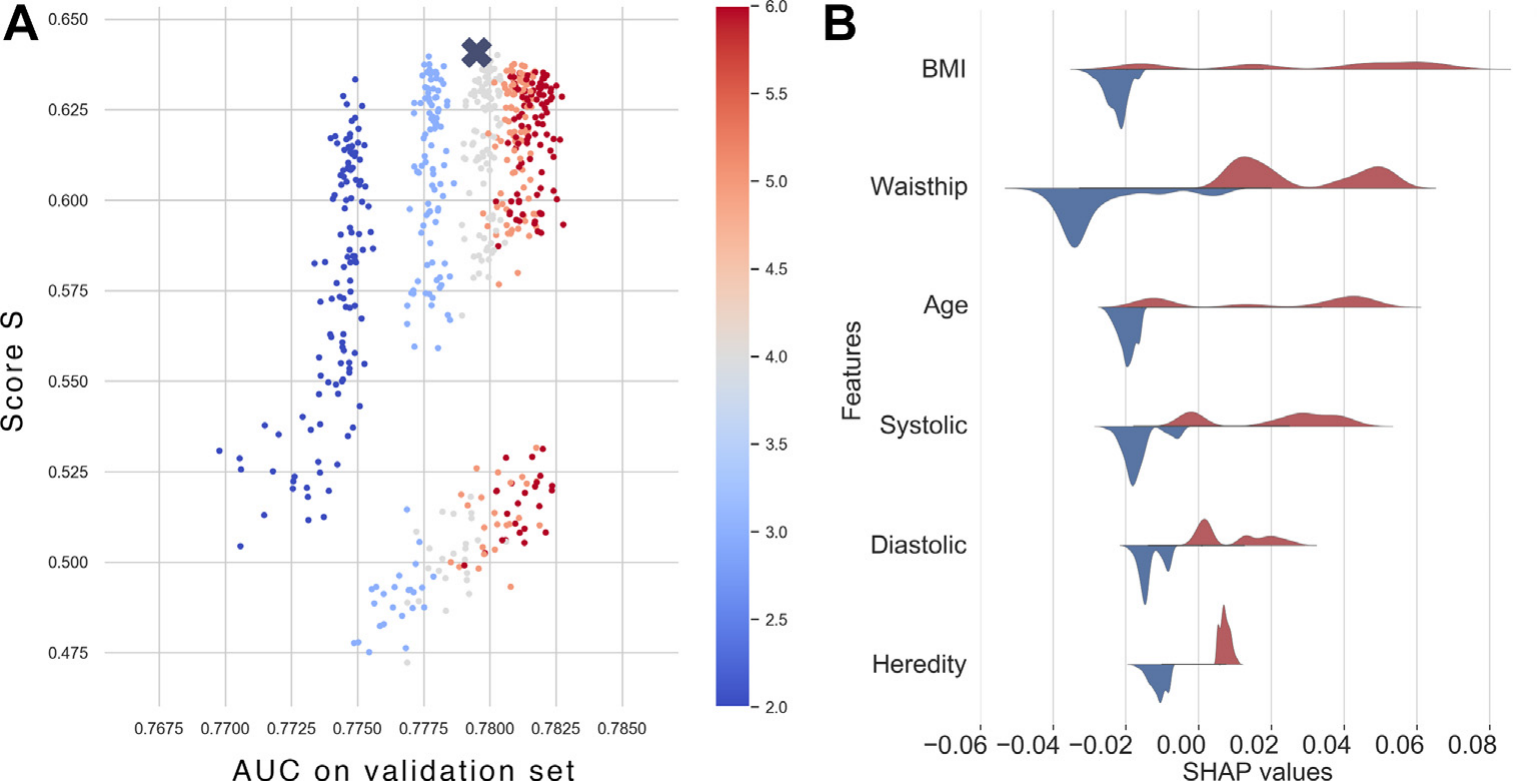
(A). The figure shows the score, *S,* vs. *AUC*^*val*^ (Area Under the Curve on Validation Set). (B) The feature importance shown as a split violin plot with the SHAPt values for feature values above (red) and below (blue) the mean values.

To find the optimal hyperparameters for the Random Forest classifier, a grid search was performed with different values of the hyperparameters; n_estimators, min_samples_leaf, max_depth and number_of_models. The models with the different hyperparameters are visualized in Figure 1A with regards to the score, *S*, and AUC on validation data, defined as the mean of the AUC scores from the cross validation. Each dot in the scatterplot represents a model with the different hyperparameters tested in the grid search. The best performing model according to *S* is marked with a cross in the figure. The color scheme in the figure show the different values of max_depth ranging from 2 to 6. The grid search and cross validation resulted in the most optimal model parameters as follows below

n_estimators: 120; min_samples_leaf 125; max_depth 4; number_of_models 30

### 4.2 SHAP summary plot

The feature importance with the 6 features that have the largest effect on the model output is shown in a SHAP summary plot in Figure 1B. The split violin plot illustrates the difference in SHAP values for feature values larger (red) and smaller (blue) than the mean for the feature. In general, feature values above the mean for the 6 features have larger SHAP values and thus indicate an increased probability for developing diabetes.

### 4.3 SHAP dependence plot

**Figure 2:**
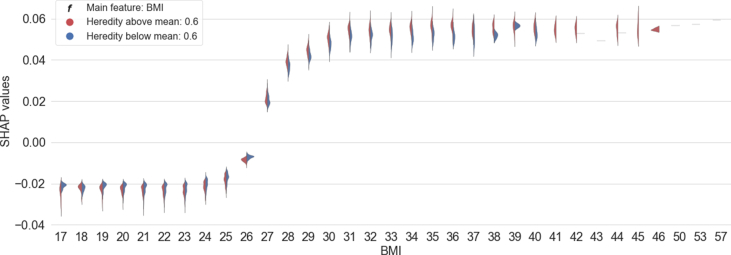
The difference in SHAP values for different BMI (body mass index, expressed as kg/m^2^) in relation to diabetes heredity. Feature values larger or smaller than the mean of the feature are depicted in red or blue color, respectively.

A SHAP dependence plot illustrates the SHAP values for different values of a feature and in addition the difference in SHAP values split on large and small values of another feature for each feature value. Figure 2 shows the feature dependence for different values of BMI with and without heredity as a split violin plot for each feature value. The figure indicates that individuals with a BMI >26 have a higher risk of developing prediabetes or T2D. In addition, the figure indicates that a combination of high BMI and heredity results in a markedly increased risk as compared to high BMI alone.

### 4.4 Individual risk profiles

**Figure 3:**
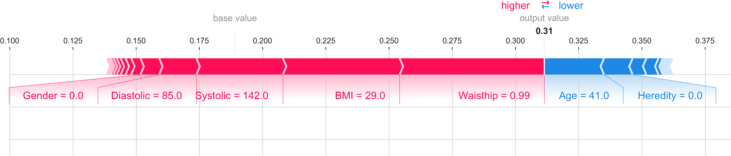
A SHAP force plot for a person in the data set with a higher risk than average to develop type II diabetes. Features depicted in red color represent higher risk, while features in blue color lower risk of diabetes.

The SHAP force plot was used to illustrate risk profiles for individuals. The *output value* indicates a person's risk to develop prediabetes or diabetes in 10 years. The *base value* shows the mean risk for the dataset. The force plot shows the features that has the largest impact on the patients predicted risk. Figure 3 shows the values for a person predicted to develop T2D. The red part of the graph illustrates the features that increase the risk and the blue part shows the features that decrease the risk of developing T2D.

**Figure 4:**
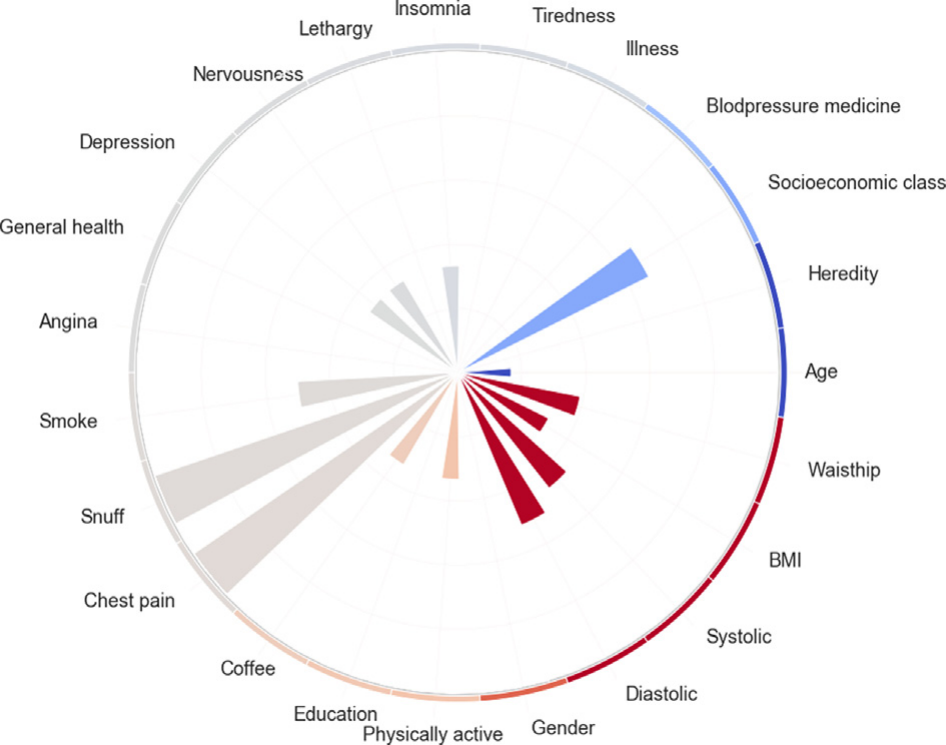
The circular bar plot shows the feature values for a person in the data set with higher than average diabetes risk. The features are colorized according to SHAP values, i.e. red indicates increased, blue decreased and grey non-significant effect on the risk.

To show features not visible in the SHAP force plot a circular bar plot was created to visualize the features, their size, and their SHAP values. The height of the bars illustrates the relative size of the feature value for each person from low to high. The color of the bars shows the SHAP values from dark red that includes the highest risk features to dark blue with the lowest risk features. The grey bars show features with minimal effect on the model outcome. Figure 4 shows the circular bar plot for the person displayed in Figure 3. The features, or factors, with putative impact on T2D risk included in the machine learning analysis are listed in Table 2.

## 5 Discussion

### 5.1 Personalized healthcare

The presented results of applying the interpretable ML models can be useful to healthcare providers for planning, operating and following up the care unit strategies and improvement programs by

1. Analyzing the patient population, or its subgroups, with respect to risk factors for developing T2D;
2. Developing treatment programs for the patient population, addressing current risk factors;
3. Following up on how well the care program work by re-training the ML model and extracting the figures on a regular basis;
4. Operating improvement work in applied health care programs and competence development within the care unit that addresses patients'current needs.

Since most of the factors affecting diabetes risk also impact the metabolic control in diabetes, the individual risk profiles can be used in the dialogue with the individual patient to develop individual care plans, and follow up and improve them based on the individual's needs and conditions. We believe that this would increase the patients' motivation to be engaged in their own care to reach the health care goals. As the analyzed research data are largely similar to those in real medical records in Sweden, it is likely that the data extracted from medical records would generate similar results. However, the time perspective on the risk of developing type 2 diabetes could be set to a shorter time that 10 years.

### 5.2 Model optimization

By optimizing the model towards the combined measure, *S*, instead of optimizing towards AUC we sacrifice some of the accuracy of the model. If the explanations of a ML model with high accuracy are not robust, the model generates inaccurate predictions. It is thus only ML models with robust interpretations that are reliable for our applications.

The ML model should find the same explanatory pattern time after time when training. Patterns that the model repeatedly finds are more accurate than those found only a few times. Our definition of robustness is a measure of how often the interpretation model finds the same patterns by measuring the standard deviation of the explanations with repeated training of the same model with somewhat different data. The risk of wrong patterns repeating themselves is lower with a larger amount of data.

Studies have been done in the past to predict the risk of developing T2D using different machine learning (ML) methods. Simple classification models (Naïve Bayes, Logistic Regression) [18, 19] have been utilized on a US dataset found on UCI's ML portal [20]. That data set contained data from 768 women older than 21 years, including the number of pregnancies of each women. Another study from China was performed on a larger amount of data using neural networks but without the use of an interpretation model [21]. In the study by Hathaway et al., the interpretable ML model SHAP was implemented to explain models predicting diabetes [22]. The major difference is that in our study we have a data set containing more than 8000 persons, while in the latter investigation data from 50 patients were studied.

The rather wellknown Findrisk questionnaire was constructed to evaluate risk of T2D and prediabetes in Finland [23]. Similar to our instrument, Findrisk evaluates the 10-year risk of illness and is also based on information on most of the features, but not all that we are using. Thus, Findrisk includes the following features: age, BMI, waist circumference, exercise, intake of vegetables and fruit, ever had drugs for hypertension treatment, ever told by physician that blood glucose was too high, family history of diabetes. Our study includes all these features, and in addition we register actual values of systolic and diastolic blood pressure and tobacco use that are well known as diabetes risk factors. We also looked at features indicating psychological distress, i.e. insomnia, depression, nervousness and fatigue, all factors that have been linked to diabetes risk in several epidemiological studies [4]. However, as shown in Figure 4, these self-reported stress factors were not of great importance in relation to the more wellknown risk factors.

## 6 Conclusions

The results from the machine learning and the interpretable model on SDPP data generated the features with the largest effects on predicting a prediabetes or T2D diagnosis 10 years after the data collection. The features with the largest effect on the outcome were BMI, waist-hip ratio, age, systolic and diastolic blood pressure, and heredity (i.e. family history of T2D). High values of the features resulted in an increased risk for developing T2D. A considerable risk for a combination of two features was shown for high BMI and heredity as compared to high BMI only.

Individual risk profiles were produced to show the risk of developing T2D for each person and visualize comprehensibly the features and values with the largest influence. For each person we could show the features and values that increase and decrease the risk compared to the mean risk in the dataset. The size of the risk of each feature can be seen in the individual risk profile. In addition, a circular bar plot was created to show the features, values, and their influence on the risk for a larger number of features.

Since most features affecting diabetes risk also play a role for metabolic control in diabetes, e.g. factors like BMI, diet, tobacco use, psychosocial factors, stress, we propose that the tool can also be used in diabetes care to develop and follow-up more individualized health care plans. Hence, we have initiated a clinical investigation with T2D patients in the primary health care, using the machine learning algorithm on data available in the medical records, to see if the results can be of value in attempts to improving diabetes care, especially when focusing on lifestyle issues.

## Declarations

### Author contribution statement

L. Lama: Conceived and designed the experiments; Performed the experiments; Analyzed and interpreted the data; Wrote the paper.

O. Wilhelmsson: Conceived and designed the experiments; Performed the experiments.

E. Norlander, P. Tynelius, L. Wärvik: Conceived and designed the experiments; Performed the experiments; Analyzed and interpreted the data.

L. Gustafsson, A. Lager: Conceived and designed the experiments.

C. -G. Östenson: Conceived and designed the experiments; Analyzed and interpreted the data; Contributed reagents, materials, analysis tools or data.

### Funding statement

This work was supported by 10.13039/501100004348Stockholms Läns Landsting (1988/10) and 10.13039/501100001858Vinnova.

### Data availability statement

Data will be made available on request.

### Declaration of interests statement

The authors declare no conflict of interest.

### Additional information

No additional information is available for this paper.
